# Cuticular Hydrocarbon Trails Released by Host Larvae Lose their Kairomonal Activity for Parasitoids by Solidification

**DOI:** 10.1007/s10886-021-01310-w

**Published:** 2021-09-16

**Authors:** Sarah Awater-Salendo, Dagmar Voigt, Monika Hilker, Benjamin Fürstenau

**Affiliations:** 1 Federal Research Centre for Cultivated Plants, Institute for Ecological Chemistry, Plant Analysis and Stored Product Protection, Julius Kühn Institute, Königin-Luise-Str.19, 14195 Berlin, Germany; 2 Dahlem Centre of Plant Science, Institute of Biology, Applied Zoology/Animal Ecology, Freie Universität Berlin, Haderslebener Str.9, 12163 Berlin, Germany; 3 Institute of Botany, Faculty of Biology, Technische Universität Dresden, Zellescher Weg 20b, 01217 Dresden, Germany

**Keywords:** Bethylidae, Insect cuticle, Perception, Trail-following behavior, Tenebrionidae, *Tribolium confusum*

## Abstract

Successful host search by parasitic wasps is often mediated by host-associated chemical cues. The ectoparasitoid *Holepyris sylvanidis* is known to follow chemical trails released by host larvae of the confused flour beetle, *Tribolium confusum*, for short-range host location. Although the hexane-extractable trails consist of stable, long-chain cuticular hydrocarbons (CHCs) with low volatility, the kairomonal activity of a trail is lost two days after release. Here, we studied whether this loss of kairomonal activity is due to changes in the chemical trail composition induced by microbial activity. We chemically analyzed trails consisting of hexane extracts of *T. confusum* larvae after different time intervals past deposition under sterile and non-sterile conditions. GC-MS analyses revealed that the qualitative and quantitative pattern of the long-chain CHCs of larval trails did not significantly change over time, neither under non-sterile nor sterile conditions. Hence, our results show that the loss of kairomonal activity of host trails is not due to microbially induced changes of the CHC pattern of a trail. Interestingly, the kairomonal activity of trails consisting of host larval CHC extracts was recoverable after two days by applying hexane to them. After hexane evaporation, the parasitoids followed the reactivated host trails as they followed freshly laid ones. Cryo-scanning electron microscopy showed that the trails gradually formed filament-shaped microstructures within two days. This self-assemblage of CHCs was reversible by hexane application. Our study suggests that the long-chain CHCs of a host trail slowly undergo solidification by a self-assembling process, which reduces the accessibility of CHCs to the parasitoid’s receptors as such that the trail is no longer eliciting trail-following behavior.

**Supplementary Information:**

The online version contains supplementary material available at 10.1007/s10886-021-01310-w.

## Introduction

Successful foraging behavior of parasitic wasps depends on recognition of host-associated cues indicating the presence and location of a potential host. On a long range, highly volatile feeding- or oviposition-induced host plant odors, as well as volatile cues released by the host itself, may guide host searching parasitoids (Hilker and McNeil 2008; Steidle and Van Loon 2003; Vinson 1998). On a short range, chemicals of low volatility may become important for host location (Colazza et al. 2014; Vinson 1998).

Chemical trails left by host insects on the plant surface or feeding substrate were shown to consist of low volatile, long-chain cuticular hydrocarbons (CHCs) (Colazza et al. 2007; Fürstenau and Hilker 2017; Rostás and Wölfling 2009). Several parasitoid species are known to recognize and follow host trails (Colazza et al. 2009; Howard et al. 1998; Lo Giudice et al. 2011; Rostás and Wölfling 2009). These trails are used as kairomones for host location, for host recognition, and even for host gender discrimination (Borges et al. 2003; Gomes Lagôa et al. 2020; Howard and Flinn 1990; Salerno et al. 2009). In addition to trails deposited by hosts, parasitoids also react to trails released by predatory insects and by conspecifics. They respond to trails left by predators by avoidance behavior (Nakashima et al. 2004) and exploit trails left by conspecifics for mate finding (Bernal and Luck 2007; Kapranas et al. 2013).

CHCs of host insects chemically make up the dominant part of trails used by parasitoids (Blomquist and Bagnères 2010; Geiselhardt et al. 2009; Gomes Lagôa et al. 2020). Insects release their CHCs onto the substrate, which is in contact with their whole body or only their tarsi (e.g., Hasenfuss 1977; Geiselhardt et al. 2009, 2010; Gerhardt et al. 2016; Lo Giudice et al. 2011; Rostás and Wölfling 2009). In general, CHCs have several important functions in insects, ranging from protection against desiccation to the mediation of intra- and interspecific communication (e.g., Gibbs 1998; Howard and Blomquist 2005; Lockey 1988; Menzel et al. 2017; Otte et al. 2018). The multifunctionality of CHCs is based on the various types of CHCs, which can be saturated or unsaturated, linear or methyl-branched (Blomquist and Bagnères 2010; Blomquist and Ginzel 2021; Gibbs 2002). According to Menzel et al. (2019), the epicuticular layer of hydrocarbons forms a solid-liquid mixture over a broad range of temperatures due to the different melting temperatures of CHC types. Thus, the physical traits of CHCs vary with fluctuating environmental conditions to maintain the biological function of CHCs (Beament 1958; Menzel et al. 2017; Sprenger et al. 2018).

The persistence of kairomonal activity of host insect trails mediating the foraging behavior of parasitoids is limited in time, despite the low volatility of CHCs. Previous studies have shown that the kairomonal effect of host CHC trails on foraging larval parasitoids decreased significantly one day after trail deposition and lasted only for a maximum of three days (Fürstenau and Hilker 2017; Rostás and Wölfling 2009). The persistence of trails laid by social insects for recruitment of nestmates to resources is usually achieved by renewal of the trail as long as the resource is available. Thus, trails of social insects consisting of more volatile compounds than long-chain CHCs can persist for a long time but would decay within minutes if not consistently renewed (Jeanson et al. 2003; Morgan 2009; Robinson et al. 2008). However, trails left by non-social host insects of parasitoids are only renewed when the host is moving by chance on the same track where it has been before.

It is unknown so far which factors contribute to the low persistence of the kairomonal effect of host insect trails on foraging parasitoids. Microbes present on the substrate or released by host insects when depositing the trail might contribute to a change in the chemical profile of trails. Microbes living in symbiosis with insects are well known to shape the production of CHCs of their hosts (Engl and Kaltenpoth 2018; Sprenger and Menzel 2020). Entomopathogenic fungi (e.g., *Beauveria bassiana* ([Bals.-Criv.] Vuill., Cordycipitaceae) and *Metarhizium anisopliae* ([Meschn.] Sorokin, Clavicipitaceae)) are known to metabolize CHCs as a carbon source for their growth, thereby alternating the CHC profiles of infected host insects (Lecuona et al. 1991; Napolitano and Juárez 1997; Pedrini et al. 2013). However, no knowledge is available on whether microorganisms are involved in the decrease of kairomonal activity of host insect trails consisting of CHCs.

Here, we addressed this gap in knowledge by studying the persistence of the chemical composition of CHC trails released by *T. confusum* (Du Val 1863) (Coleoptera: Tenebrionidae), under sterile conditions (excluding microbial activity) and non-sterile ones. The CHC trails left by larvae of this beetle are followed by the larval ectoparasitoid *Holepyris sylvanidis* (Bréthes 1913) (Hymenoptera: Bethylidae), which attacks larvae of several stored-product beetles, including different species of the genus *Tribolium* (Amante et al. 2017, 2018; Evans 1969; Fürstenau and Hilker 2017)*. Holepyris sylvanidis* discriminates between a host and a non-host species by a specific pattern of methyl-branched alkanes. The host-specific CHC pattern is common to the cuticle of different host beetle species (Awater-Salendo et al. 2020). Hexane extracts of host larvae deposited as trails elicit trail-following behavior of *H. sylvanidis*. The kairomonal effect of *T. confusum* larval trails on *H. sylvanidis* females was shown to last for at maximum two days, although these trails are exclusively composed of long-chain, low volatile, saturated CHCs (Fürstenau and Hilker 2017).

In detail, our study investigated the following questions by analyzing CHC trails of *T. confusum* larvae extracted with hexane: (1) Does the chemical composition of trails change over time past trail deposition when microbial degradation is excluded? (2) Does the chemical composition of trails from host larval extracts, which are not excluded from microbial degradation, change over time past trail deposition? Therefore, we analyzed the quantitative and qualitative CHC profiles of sterile-filtered and non-filtered CHC trails 0 h, 24 h, and 48 h after deposition by coupled gas chromatography-mass spectrometry (GC-MS). Furthermore, we asked (3) whether the kairomonal activity of 48 h-old, inactive trails from host larval extracts can be recovered by the application of hexane and (4) whether changes in physical structures of trails from host larval extracts 1 h and 48 h past deposition can be visualized by cryo-scanning electron microscopy (cryo-SEM).

## Methods and Material

### Insects

Individuals of *H. sylvanidis* and *T. confusum* were taken from a permanent rearing maintained at the Institute for Ecological Chemistry, Plant Analysis and Stored Product Protection (Julius Kühn Institute, Berlin, Germany). The insects were reared on wheat grist according to a protocol described by Fürstenau et al. (2016). For chemical analyses of host larval trails, we used 4th instar *T. confusum* larvae, which represent the preferred host stage of the parasitoid (Awater-Salendo et al. 2020). For the trail-following bioassays, we used unmated, one- to five-day-old *H. sylvanidis* females without previous oviposition experience.

### Preparation of CHC Extracts from *T. confusum* Host Larvae

Since hexane extracts of *T. confusum* larvae elicit trail-following behavior in female *H. sylvanidis* when freshly applied onto a substrate but lose their kairomonal activity after two days (Fürstenau and Hilker 2017), we here studied whether the chemical composition of these trails changes over time past deposition. We used hexane extracts of 4th instar larvae (hereafter referred to as “larval trails”) instead of naturally laid trails by larvae for our analyses because thus the quantities of compounds detected in the trails could be exactly determined and referred to larval individuals (larval equivalents = LE per volume hexane; see below).

The chemical composition of larval trails was analyzed 0 h, 24 h, and 48 h after trail deposition on glass Petri dishes (for details, see below). We analyzed freshly laid trails (0 h), i.e., the trails were extracted from the substrate immediately after deposition, and “aged” trails (24 h, 48 h), i.e., the trails were extracted from the substrate 24 h and 48 h after deposition. During these time intervals (24 h, 48 h), the trails were kept at room temperature and approximately 30.5 to 35.2% RH. In addition to the question of whether the chemical composition of trails changes over time past deposition, we also investigated whether the in- and exclusion of microbes affect the chemical trail composition. For GC-MS analyses, we, therefore, prepared (1) hexane extracts, which were sterile-filtered, and (2) non-sterile-filtered extracts. For trail-following bioassays with *H. sylvanidis* females and microscopic imaging of larval trails, we prepared non-sterile-filtered larval extracts (3).

For the preparation of stock solutions, the number of *T. confusum* larvae extracted varied according to their availability. However, we extracted a pool of larvae with always a defined number of individuals per microliter. This allowed us to calculate the exact number of LE per microliter finally deposited per trail. For all extracts (stock solutions), larvae were first killed by freezing them at −20 °C for up to 30 min and then thawed for ca. 2 min at ambient temperature. Thereafter, larvae were extracted by gentle shaking them for 10 min in *n-*hexane (analytical purification >98%, VWR, Radnor, USA). By this procedure, the layer of superficial CHCs is removed from larval integuments and dissolved in hexane (Fürstenau and Hilker 2017). The supernatant was further processed. The detailed protocols for the preparation of the above-mentioned three types of extracts are provided below. All samples prepared for GC-MS analysis, bioassays, or microscopic imaging were stored at −20 °C prior to usage.

- (1) Sterile *T. confusum* hexane extracts for chemical analysis. The preparation of these extracts aimed to exclude any possible effects of microorganisms originating from the host (e.g., microbes on the cuticle of *T. confusum* larvae), the host habitat (here: wheat grist), or the environment (e.g., airborne microorganisms) on the CHC composition of host trails after different time intervals.

In total, five stock solutions were prepared. The supernatant was removed from the stock solution with a disposable syringe and loaded onto a sterile PVDF filter (0.22 μM, Carl Roth, Karlsruhe, Germany), which had been pre-conditioned by rinsing it with 10 ml *n*-hexane. Syringes were cleaned three times with *n*-hexane before being used. The PVDF filter loaded with larval extract was eluted with *n*-hexane. Sterile-filtered extracts were then concentrated to dryness under a gentle stream of nitrogen, dissolved in *n*-hexane, and frozen at −20 °C until further processing.

Under a clean bench, a trail of the sterile-filtered hexane extract was applied to the periphery of the bottom part of a glass Petri dish (diameter: 90 mm). The dish had previously been cleaned with demineralized water and 70% ethanol solution (analytical purification >96.0%, Berkel AHK, Ludwigshafen, Germany) and subsequently sterilized at 175 °C for 3.5 h. After trail application, the solvent was allowed to evaporate for 1 min. We prepared five replicates (Petri dishes, *N* = 5) for each investigated time interval past trail deposition. For the 0 h interval, trails were re-extracted in *n*-hexane immediately after deposition, transferred to a 2 ml vial, evaporated to dryness under a gentle stream of nitrogen, and then dissolved in a distinct volume of *n*-hexane with 1-eicosene as an internal standard (IS, Sigma-Aldrich, Taufkirchen, Germany). For trails to be investigated 24 h and 48 h after trail deposition, the dishes were sealed with Parafilm® and kept in a closed sterile box outside the sterile bench. After 24 h and 48 h storage, trails were removed from the dishes as described for the immediately analyzed trails.

- (2) Non-sterile *T. confusum* hexane extracts for chemical analysis. To imply possible effects of host-associated or environmentally present microorganisms on the CHC composition of host trails at different time intervals past trail deposition, larval hexane extracts were prepared under non-sterile conditions.

In total, we prepared six stock solutions. In contrast to the sterile-filtered extracts, here the supernatants were not filtered but directly concentrated to dryness under a gentle stream of nitrogen, dissolved in *n*-hexane, and frozen at −20 °C for further analysis.

As described for the sterile extracts, trails were deposited on eighteen Petri dishes (*N* = 6 per time interval) which had been cleaned but not sterilized by heat prior to application. After solvent evaporation, Petri dishes were kept open under a laboratory hood for 0 h, 24 h, or 48 h (non-sterile conditions). After the respective time intervals, larval trails were re-extracted in *n*-hexane and quickly transferred to a vial. These extracts were then concentrated under a gentle stream of nitrogen to dryness, and re-dissolved in a distinct volume of *n*-hexane containing again 1-eicosene as internal standard.

- (3) Non-sterile *T. confusum* hexane extracts for bioassays and microscopic imaging. We studied the parasitoid’s behavioral responses to larval trails in dependence (a) of the time intervals past deposition (“untreated trails”) and (b) of re-dissolving “aged” trails by *n-*hexane (“re-dissolved trails”) (see below for bioassay method). For each type of trail, we prepared seven non-sterile-filtered stock solutions (*N* = 7).

For cryo-SEM (see below), we took the same samples from the stock solutions of larval extracts.

### GC-MS Analysis of Sterile- and Non-sterile-Filtered *T. confusum* Host Larval Trails

Samples were analyzed on a 6890 N GC coupled to a 5975 B VL MS quadrupole mass spectrometer (Agilent Technologies, Waldbronn, Germany). As carrier gas, we used helium with a flow rate of 1.1 ml min^−1^ and a fused silica column (HP-5MS capillary column, 30 m × 0.25 mm × 0.5 μm, Agilent Technology, Waldbronn, Germany) as a stationary phase. One μl of each sample was injected at 250 °C in splitless mode. The oven temperature program started at 40 °C for 4 min, then raised by 10 °C min^−1^ up to 300 °C, which was held for 10°min. After a solvent delay of 5 min, the detector scanned 4.45 times s^−1^ for fragments in a range from 35 to 500 *m/z* (electron impact [EI] ionization = 70°eV).

Linear alkanes were identified by comparing their retention indices (RIs) and mass spectra with those of an authentic *n*-alkane standard (*n-*C7 - *n-*C40, Sigma Aldrich, Taufkirchen, Germany). Since no reference standards of methyl-branched alkanes were available to us, we tentatively identified the detected ones based on their characteristic mass spectrometric fragmentation pattern and RIs calculated according to Van den Dool and Kratz (1963). Additionally, RIs and fragmentation pattern of these substances were compared to those published by Fürstenau and Hilker (2017). Individual compounds (linear and methyl-branched alkanes) were quantified relative to the peak area of the IS (1-eicosene).

### Trail-Following Bioassays

To analyze the trail-following responses of *H. sylvanidis* females to *T. confusum* larval trails, we used a “walking arena”, i.e. the lower part of a glass Petri dish (diameter = 190 mm). We modified the experimental set-up described by Fürstenau and Hilker (2017) as follows. The rim of each Petri dish was coated with an aqueous solution of Teflon (Sigma Aldrich, Taufkirchen, Germany) to prevent parasitoids from climbing up during the bioassays. Below the bottom of the dish, we attached a drawing of two circles (diameter: 80 mm each) as a template for the trails (one test and one control trail) to be laid on the glass side. A test trail consisted of a hexane extract of *T. confusum* larvae, while a control trail consisted of only hexane. The two circles were 30 mm apart from each other, and they were about 3 mm wide on the periphery. Additionally, each circle was divided into eight equally sized sections (length: 31 mm each). A strip of light-emitting diodes (λ = 625 nm, Barthelme GmbH & Co, Nuremberg, Germany) was located 300 mm above the arena for consistent illumination.

We tested the trail-following responses of *H. sylvanidis* females to two different types of larval trails: (1) larval trails at different time intervals after deposition (0 h, 24 h, and 48 h); (“untreated trails”); and (2) larval trails at different time intervals after deposition (0 h and 48 h); these trails had been treated with *n*-hexane just prior to exposure to the parasitoids (“re-dissolved trails”). All bioassays were performed at room temperature and approximately 35 to 40% RH on three consecutive days.

When testing untreated trails, we deposited 25 μl (corresponding to 5 LE) of a stock solution on a test circle in the Petri dish. We knew from our previous study that *H. sylvanidis* shows trail-following behavior already to very low-concentrated larval extracts (Fürstenau and Hilker 2017). The control trail consisted of 25 μl *n*-hexane. Each stock solution was used once for each time interval and type of trail (*N* = 7). Host larval trails tested at later time intervals after deposition were kept under a laboratory hood in open Petri dishes for 24 h or 48 h prior to the beginning of the respective bioassays. A bioassay started after releasing one *H. sylvanidis* female at a randomly selected position between the circular control and test trail and lasted 300 s.

Since the results of the bioassay with “untreated trails” revealed that differences in the parasitoid’s response were the greatest between 0 h and 48 h after trail deposition, we decided to use only these two time intervals in the bioassay with “re-dissolved trails”.

When testing the “re-dissolved trails”, test and control trails were first applied to the dishes as described for “untreated trails”. Thereafter, just prior to testing, 25 μl additional *n*-hexane was applied on the circular test and the control trail. The hexane was allowed to evaporate for 1 min. The bioassay started then by releasing one *H. sylvanidis* female into the walking arena as described above.

When parasitoid females exhibited trail-following behavior, they intensely antennated host larval trails and followed them in a zigzag line (Howard and Flinn 1990). *Holepyris sylvanidis* females also tended to reverse the walking direction or to stop following a trail to explore the local area before returning to the trail. Therefore, we evaluated the behavioral response of *H. sylvanidis* to each treatment based on two parameters: (1) the distance covered by a parasitoid was quantified by the number of sections, which a parasitoid fully walked on test and control circles; (2) the total time spent by a parasitoid on each trail was recorded using the computer software “The Observer 3.0” (Noldus Information Technology, Wageningen, The Netherlands). After each run, we replaced the tested individual and changed the position of control and test circle to avoid any biased results due to possible side preferences. After having tested four parasitoids, the bioassay arena was replaced by a new one. When freshly applied trails (= 0 h) were tested, we reused the test arena after cleaning it with demineralized water and a 70%-ethanol solution. Parasitoids, which rested more than 50% of the observation time, were excluded from the statistical analysis and replaced by a new one. This was the case in less than 4% (5 occasions) of all experiments. In total, we tested each type of trail at each time interval past deposition with 28 female *H. sylvanidis* (*N* = 28).

### Cryo-SEM

To study possible changes in physical structure of CHC-consisting trails at different time intervals past trail deposition, the cryo-SEM SUPRA 40VP-31-79 (Carl Zeiss SMT Ltd., Oberkochen, Germany) equipped with an EMITECH K250X cryo-preparation unit (Quorum Technologies Ltd., Ashford, Kent, UK) was used.

Hexane extracts of host larvae were applied onto either a polar substrate or a non-polar one. We used the polar substrate because (i) we here always applied host larval trails on glass (polar) when studying the parasitoids’ behavioral response to them and (ii) *T. confusum* might encounter polar substrates in nature (e.g., fine flour with its carbohydrates). We used also an apolar substrate because these substrates might also be present in the natural habitat of *T. confusum* larvae (e.g., waxy seed coats). The polar substrate consisted of 5 × 5 mm pieces of ultra-flat silicon wafer thermically covered with a 200 nm thick, polished SiO_2_ film (surface roughness: 2–3 Å) (Plano GmbH, Wetzlar, Germany). The substrate had been cleaned prior to use by successive immersions in Piranha solution (mixture of sulphuric acid H_2_SO_4_ and hydrogen peroxide H_2_O_2_, 3:1), rinsed with distilled water, and dried by compressed air. To obtain apolar substrates, which are known to promote wax crystallization (e.g., Niemietz et al. 2009), we silanized the polar substrate with 1H,1H,2H,2H perfluorodecyltrichlorosilane (C_10_H_4_Cl_3_F_17_Si, 97%, SIH5841.0, ABCR GmbH & Co. KG, Karlsruhe, Germany).

The CHC larval extract was slowly applied in 5 μl steps onto the substrate. In total, 25 μl could be placed onto the small piece of substrate. The extracts were kept for 1 h or 48 h on the substrates at ambient temperature and 25.7 ± 2.79% RH until further processing (“untreated trails”). In addition, we applied a droplet of hexane to samples with larval extracts, which had been on the substrate for 1 h or 48 h (“re-dissolved trails”).

For cryo-SEM analysis, the substrates were mounted on metal stubs using polyvinyl alcohol (Tissue-Tek, OCT, Sakura Finetek Europe BV, Alphen aan den Rijn, the Netherlands). The solvent was evaporated for at least 1 h to avoid interferences inside the cryo-SEM. Subsequently, the samples were shock-frozen in liquid nitrogen in the slushing chamber, transferred to the cryo-preparation chamber at −140 °C, sublimed for 15 min at −70 °C, sputter-coated with platinum (layer thickness ca. 10 nm), transferred to the SEM, and then examined in a frozen state at 5 kV accelerating voltage and − 100 °C temperature. Cryo-SEM micrographs were taken using the software Smart SEM 05.03.05 (Carl Zeiss SMT Ltd., Oberkochen, Germany).

### Statistical Analysis

We conducted all statistical analysis using R, version 4.0.2 (R Core Team 2020), except for the analysis of similarity percentages (SIMPER), which was performed in “PAST”, version 3.26 (Hammer et al. 2001).

For statistical comparison of the quantitative chemical pattern of sterile- or non-sterile-filtered trails, we selected the analyzed compounds by the following criteria: (i) presence in more than 50% of all samples taken per time interval past deposition; (ii) peak area larger than 0.01% of the total peak area. If a compound was meeting criterion (i), but not criterion (ii) (i.e., the compound was below the detection limit), the “in some samples, missing value” was handled as follows. We used the “*rnorm()*”-function in R to generate a random peak for each missing value. We selected the smallest peak area, which the missing value had in other samples of the same time interval and trail type (sterile- or non-sterile-filtered), as mean and calculated the standard deviation based on the three smallest peak areas. To avoid any bias in the subsequent statistical analyses, nine pseudo-peaks were generated in the samples of sterile-filtered larval trails, while one pseudo-peak was used for non-sterile-filtered larval trails. We normalized the peak areas of selected compounds by referring the quantity (peak area) of each compound to the IS and then to one larval equivalent (LE).

To assess whether the chemical composition of sterile-filtered larval CHC trails varied quantitatively over time, quantities of each selected compound were compared by a one-way ANOVA when data were normally distributed. If the *Shapiro-Wilk* test of normality revealed that data of some compounds were not normally distributed at all time intervals, the *Kruskal-Wallis* test was computed instead, followed by *Wilcoxon’s* rank-sum test with *Bonferroni-Holm* correction. Furthermore, the dissimilarity in the chemical composition of these larval trails was tested by running a one-way analysis of similarity (ANOSIM). For this purpose, we calculated the relative amounts of detected compounds in one LE per time interval and summed them up to 100%. Based on *Bray-Curtis* dissimilarity, an ANOSIM was performed with 99,999 random permutations using the package “vegan” (version 2.5–6, Oksanen et al. 2019) in R. The dissimilarity of groups is stated by the *R*-value; groups with an *R*-value close to 0 are highly similar, while groups with an *R*-value close to 1 can be clearly discriminated (Clarke 1993). For visualization of results obtained by the ANOSIM, a non-metric multidimensional scaling (NMDS) was calculated based on the *Bray-Curtis* dissimilarity. To evaluate how well the algorithm of NMDS fits in the used data set, we calculated the associated stress value. According to Dexter et al. (2018), a stress value <0.1 indicates that the applied NMDS is a good representative. A SIMPER was implemented to identify compounds that contributed the most to the dissimilarity. Likewise, the statistical analysis of trails from non-sterile-filtered larval extracts was performed.

For each time interval, the quantitative chemical compositions of sterile- and non-sterile-filtered larval CHC trails were statistically analyzed by using a *Student’s t-* test for independent data. When the variance was unequal in both data sets, a *Welch’s t*-test was applied instead. If data of one CHC trail type were not normally distributed according to the *Shapiro-Wilk* test of normality, we used *Wilcoxon’s* rank-sum test for independent data.

The behavioral responses of *H. sylvanidis* to host larval trails were statistically analyzed (a) by comparing the walking distances and residence times on test circles with the CHC trail and on control circles with the solvent. These parameters were statistically compared by a *Student’s t*- test for paired samples. If data sets were not normally distributed according to the *Shapiro-Wilk* test, we performed a *Wilcoxon’s* signed-rank test for paired data. Furthermore, we compared (b) whether walking distances and residence times on test circles differed when trails were offered 0 h, 24 h, or 48 h after deposition. Therefore, we determined the difference (Δ) in walking distance and residence time spent on the test and the control circle for each time interval. When analyzing the responses to “untreated test and control trails”, the differences in residence times and walking distances in bioassays with trails tested 0 h, 24 h, or 48 h after deposition were not normally distributed according to the *Shapiro-Wilk* test. Therefore, we performed a *Kruskal-Wallis* test for each parameter (walking distance, residence time) followed by a pairwise *Wilcoxon’s* rank-sum test with *Bonferroni-Holm* correction. When comparing the responses to “re-dissolved trails” on test circles 0 h and 48 h after deposition, we applied a *Student’s t-* test for each parameter (walking distance, residence time) if the data were normally distributed. If normal distribution of data was absent, a *Wilcoxon’s* rank-sum test was performed.

## Results

### No Changes in the Chemical Composition of *T. confusum* Host Larval Trails in the Course of Time

We addressed the question of whether host larval trails, which had been excluded from microbial degradation, changed their chemical composition in the course of time (0 h, 24 h, and 48 h) past deposition. Our chemical analysis revealed the same 20 compounds in all investigated sterile-filtered trails, regardless of the time past trail deposition (Table 1).

**Table 1:** Cuticular hydrocarbons identified from sterile-filtered hexane extracts Of *Tribolium confusum* larvae 0 H, 24 H OR 48 H after trail (extract) deposition. Mean amounts (NG ± SE LE^−1^) And Relative Quantities (% PER LE) are given

					Hours after trail deposition^f^						
					0		24		48		
No.^a^	Compound^b^	ID^c^	RI_cal_^d^	RI_lit_^e^	Mean ± SE (ng)^g^	(%)^g^	Mean ± SE (ng)^g^	(%)^g^	Mean ± SE (ng)^g^	(%)^g^	*P*^h^
1	*n*-C25	I	2498	2500	20.52 ± 3.04	17.22	17.99 ± 3.53	17.14	21.83 ± 2.40	17.63	ns
2	11−/13-MeC25	II	2533	2534	0.13 ± 0.03	0.11	0.13 ± 0.03	0.12	0.15 ± 0.03	0.12	ns
3	5-MeC25	III	2550	2550	0.08 ± 0.02	0.07	0.08 ± 0.02	0.07	0.10 ± 0.01	0.08	ns
4	3-MeC25	IV	2573	2571	0.40 ± 0.09	0.33	0.36 ± 0.10	0.33	0.40 ± 0.08	0.32	ns
5	*n*-C26	V	2598	2599	3.10 ± 0.43	2.58	2.61 ± 0.54	2.45	3.07 ± 0.34	2.48	ns
6	10−/11−/12−/13-MeC26	VI	2633	2632	0.53 ± 0.04	0.46	0.46 ± 0.11	0.43	0.51 ± 0.09	0.41	ns
7	4-MeC26	VII	2656	2656	0.27 ± 0.03	0.23	0.21 ± 0.06	0.21	0.22 ± 0.05	0.18	ns
8	*n*-C27	VIII	2700	2700	53.27 ± 5.70	45.07	46.66 ± 7.15	45.49	57.52 ± 5.41	46.65	ns
9	11−/13-MeC27	IX	2730	2731	8.10 ± 0.75	6.95	7.25 ± 1.51	6.91	8.61 ± 1.12	6.91	ns
10	5-MeC27	X	2747	2750	3.81 ± 0.32	3.28	3.35 ± 0.65	3.25	3.84 ± 0.45	3.11	ns
11	3-MeC27	XI	2771	2773	3.50 ± 0.39	2.97	3.07 ± 0.59	2.94	3.58 ± 0.38	2.89	ns
12	5,X-DiMeC27	XII	2778	2781	1.95 ± 0.15	1.69	1.71 ± 0.37	1.64	1.86 ± 0.27	1.50	ns
13	*n*-C28	XIII	2797	2799	4.86 ± 0.53	4.11	4.23 ± 0.71	4.11	4.97 ± 0.50	4.03	ns
14	3,X-DiMeC27	XIV	2803	2807	2.64 ± 0.18	2.28	2.15 ± 0.49	2.04	2.42 ± 0.31	1.97	ns
15	12−/13−/14-MeC28	XV	2828	2831	0.93 ± 0.07	0.81	1.07 ± 0.32	1.02	0.70 ± 0.14	0.56	ns
16	4-MeC28	XVI	2854	2856	0.54 ± 0.03	0.47	0.47 ± 0.13	0.46	0.36 ± 0.07	0.30	ns
17	*n*-C29	XVIII	2897	2904	11.77 ± 1.28	9.99	10.42 ± 1.86	10.04	11.96 ± 1.20	9.71	ns
18	11−/13-MeC29	XIX	2928	2931	1.25 ± 0.12	1.07	1.15 ± 0.29	1.06	0.93 ± 0.09	0.78	ns
19	3-MeC29	XXI	2970	2978	0.23 ± 0.05	0.20	0.27 ± 0.11	0.22	0.31 ± 0.06	0.27	ns
20	*n*-C31	XXV	3096	3100	0.13 ± 0.01	0.12	0.08 ± 0.04	0.07	0.10 ± 0.01	0.08	ns

^a^Number of peaks identified in the total ion chromatogram

^b^*n*-alkanes were identified by comparing RIs and mass spectra with authentic standards. Methyl-branched alkanes were tentatively identified by the diagnostic ions, which resulted from favored fragmentation at branched points (see by Fürstenau and Hilker 2017, and by comparing RIs with data from literature

^c^Identity of CHCs used for comparison of sterile- and non-sterile-filtered larval host trails

^d^RI_cal_ = Retention index calculated on a HP-5 ms capillary column (30 m × 0.25 mm × 0.5 μm)

^e^RI_lit_ = Retention index as reported for compounds analyzed on HP-5 ms or similar columns in the database (http://www.pherobase.com/) and by Fürstenau and Hilker (2017)

^f^For the preparation of host larval trails, see experimental part

^g^For each time interval, five replicates were used (*N* = 5)

^h^For each compound, a *p* value denotes a significant quantitative difference between sterile-filtered larval CHC trails of *T. confusum* 0 h, 24 h, and 48 h after trail deposition (one-way ANOVA or *Kruskal-Wallis* test, ns = not significant)

**Fig. 1:**
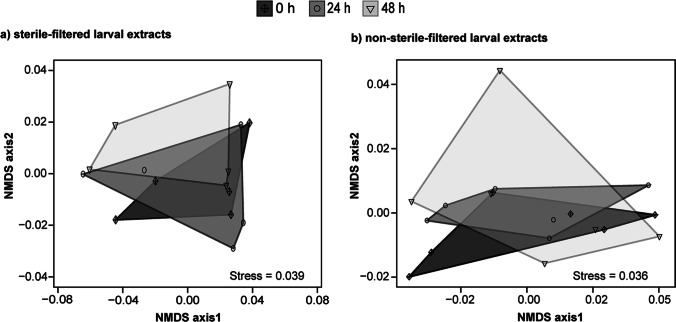
Non-metric multidimensional scaling (NMDS) visualization of the CHC composition of trails consisting of two differently treated *Tribolium confusum* larval extracts 0 h, 24 h, and 48 h after trail deposition: **a)** sterile-filtered hexane extracts, which were applied and kept under sterile conditions (*N* = 5) and **b)** non-sterile-filtered hexane extracts, which were applied and kept under non-sterile conditions (*N* = 6)

The detected trail compounds included six *n*-alkanes, twelve monomethyl-branched alkanes, and two dimethyl-branched alkanes with a chain length from C25 to C31. In all CHC profiles of larval trails and at all time intervals past trail deposition *n*-alkanes were the dominating substance group accounting for 80%, while methyl-branched alkanes accounted for about 20% (Table 1). The quantities of individual CHCs did not differ significantly between freshly laid trails (0 h), 24 h- and 48 h-old ones. Additionally, the ANOSIM confirmed that the CHC compositions of larval trails were highly similar at all time intervals past trail deposition (*R* = −0.141, *P* = 0.941). CHC profiles of these trails clustered closely in a NMDS plot calculated on the relative proportions of single compounds within one LE of *T. confusum* (Fig. 1a). According to the SIMPER analysis, *n*-C25, *n*-C27, and *n*-C29 (entries 1, 8, and 18, Table 1) contributed the most to the moderate dissimilarity (59%, Table S1). Hence, at sterile conditions, CHC profiles of freshly laid host larval trails and those, which were 24 h or 48 h old, did not significantly differ. The CHC profiles did not change within two days.

**Fig. 2:**
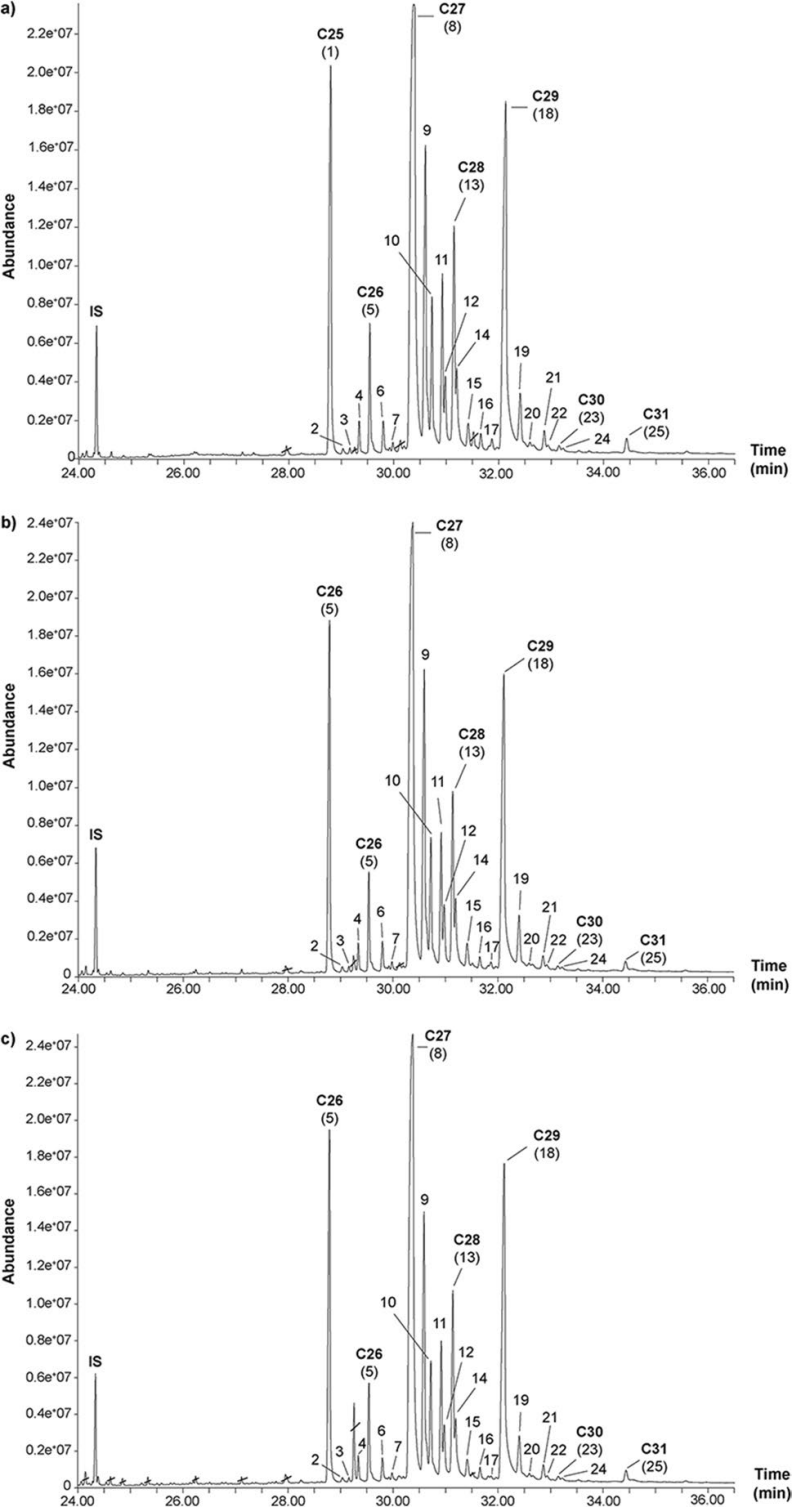
Partial total ion chromatograms (TIC) of trails consisting of non-sterile-filtered hexane extracts of *Tribolium confusum* larvae; trails (hexane extracts) were extracted from a substrate and analyzed **a)** 0 h, **b)** 24 h, and **c)** 48 h after deposition. Numbers above peaks refer to the identified compounds listed in Table 2. The *n*-alkanes (*n*-C25 – *n*-C31) detected in the larval trails are highlighted in bold. Crossed-out compounds are contaminations

We further addressed the question of whether CHC profiles of host larval trails kept under non-sterile conditions change over time. The detected compounds of non-sterile trails after the different time intervals past deposition (Fig. 2) included linear alkanes, which accounted for almost 70% in CHC profiles of these trails, and methyl-branched alkanes, which made up about 30% of the detected CHCs (Table 2). No significant differences were measured between the relative quantity of individual compounds present in freshly laid trails (0 h) and in “aged trails” 24 h and 48 h after deposition. As a result, the CHC profiles clustered closely in a NMDS plot (Fig. 1b). This finding indicated a strong similarity of the chemical composition of non-sterile-filtered larval trails analyzed after different times past deposition. This similarity was confirmed by the results of the ANOSIM (*R* = −0.144, *P* = 0.953). The SIMPER analysis revealed that *n*-C25, *n*-C27, 11−/13-MeC27, and 5-MeC27 (entries 1, 8, 9, and 10) contributed the most to the moderate dissimilarity (57%, Table S2). Thus, at non-sterile conditions, no significant differences were detected in the qualitative and quantitative CHC pattern of 0 h-, 24 h- and 48 h-old trails of *T. confusum* larvae. The long-chain CHC profiles were stable over a period of two days.

**Table 2:** Cuticular hydrocarbons identified from non-sterile-filtered hexane extracts of *Tribolium confusum* larvae 0 h, 24 h or 48 h after trail (extract) deposition. Mean amounts (ng ± SE LE−1) and relative quantities (% per LE) are given

					Hours after trail deposition^f^						
					0		24		48		
No.^a^	Compound^b^	ID^c^	RI_cal_^d^	RI_lit_^e^	Mean ± SE (ng)	(%)	Mean ± SE (ng)	(%)	Mean ± SE (ng)	(%)	*P*^g^
1	*n*-C25	I	2503	2500	20.12 ± 0.67	11.26	19.47 ± 0.77	11.41	19.10 ± 0.49	11.25	ns
2	11−/13-MeC25	II	2534	2534	0.56 ± 0.06	0.31	0.46 ± 0.03	0.27	0.63 ± 0.10	0.37	ns
3	5-MeC25	III	2550	2550	0.36 ± 0.04	0.20	0.31 ± 0.02	0.18	0.38 ± 0.06	0.22	ns
4	3-MeC25	IV	2573	2571	1.31 ± 0.12	0.73	1.22 ± 0.06	0.71	1.28 ± 0.07	0.75	ns
5	*n*-C26	V	2599	2599	5.19 ± 0.23	2.90	5.02 ± 0.15	2.95	5.12 ± 0.15	3.01	ns
6	10−/11−/12−/13-MeC26	VI	2633	2632	1.44 ± 0.16	0.79	1.37 ± 0.09	0.80	1.50 ± 0.15	0.88	ns
7	4-MeC26	VII	2658	2656	0.79 ± 0.08	0.44	0.66 ± 0.05	0.39	0.82 ± 0.10	0.48	ns
8	*n*-C27	VIII	2708	2700	56.50 ± 2.77	31.50	53.37 ± 1.68	31.28	52.23 ± 0.78	30.75	ns
9	11−/13-MeC27	IX	2735	2731	15.75 ± 1.56	8.68	15.13 ± 0.90	8.86	14.72 ± 0.95	8.62	ns
10	5-MeC27	X	2750	2750	7.61 ± 0.65	4.20	7.24 ± 0.34	4.24	8.30 ± 1.19	4.85	ns
11	3-MeC27	XI	2774	2773	7.30 ± 0.30	4.07	7.17 ± 0.10	4.21	7.02 ± 0.16	4.13	ns
12	5,X-DiMeC27	XII	2781	2781	3.79 ± 0.35	2.09	3.47 ± 0.19	2.03	3.36 ± 0.18	1.97	ns
13	*n*-C28	XIII	2801	2799	10.45 ± 0.41	5.84	10.28 ± 0.18	6.03	10.18 ± 0.21	5.99	ns
14	3,X-DiMeC28	XIV	2806	2807	5.10 ± 0.35	2.83	4.82 ± 0.16	2.82	4.80 ± 0.23	2.82	ns
15	12−/13−/14-MeC28	XV	2830	2831	1.98 ± 0.19	1.09	1.86 ± 0.12	1.09	1.87 ± 0.18	1.09	ns
16	4-MeC28	XVI	2856	2856	1.50 ± 0.12	0.83	1.29 ± 0.05	0.76	1.35 ± 0.11	0.79	ns
17	3-MeC28	XVII	2873	2872	0.71 ± 0.16	0.39	0.44 ± 0.02	0.26	0.57 ± 0.08	0.33	ns
18	*n*-C29	XVIII	2904	2904	29.10 ± 1.57	16.18	28.06 ± 0.52	16.46	27.68 ± 0.33	16.28	ns
19	11−/13-MeC29	XIX	2930	2931	4.51 ± 0.43	2.49	4.06 ± 0.32	2.38	4.10 ± 0.39	2.40	ns
20	5-MeC29	XX	2948	2948	0.91 ± 0.11	0.50	0.70 ± 0.02	0.41	0.71 ± 0.05	0.41	ns
21	3-MeC29	XXI	2972	2978	1.40 ± 0.10	0.78	1.29 ± 0.05	0.76	1.34 ± 0.09	0.78	ns
22	5,X-DiMeC29	XXII	2979	2980	0.83 ± 0.05	0.46	0.65 ± 0.03	0.38	0.70 ± 0.06	0.41	ns
23	*n*-C30	XXIII	2999	3000	0.64 ± 0.03	0.35	0.54 ± 0.03	0.32	0.58 ± 0.05	0.34	ns
24	3,X-DiMeC29	XXIV	3004	3005	0.68 ± 0.07	0.38	0.55 ± 0.04	0.32	0.61 ± 0.12	0.36	ns
25	*n*-C31	XXV	3098	3100	1.29 ± 0.04	0.73	1.15 ± 0.10	0.68	1.19 ± 0.05	0.70	ns

^a^Peak numbers referring to Fig. 2

^b^*n*-alkanes were identified by comparing RIs and mass spectra with authentic standards. Methyl alkanes were tentatively identified by the diagnostic ions, which resulted from favored fragmentation at branched points (see by Fürstenau and Hilker 2017), and by comparing RIs with data from literature

^c^Identity of CHCs used for comparison of sterile and non-sterile-filtered larval host trails

^d^RI_cal_ = Retention index calculated on a HP-5 ms capillary column (30 m × 0.25 mm × 0.5 μm)

^e^RI_lit_ = Retention index as reported for compounds analyzed on HP-5 ms or similar columns in the database (http://www.pherobase.com/) and by Fürstenau and Hilker (2017)

^f^For the preparation of host larval trails, see experimental part

^g^For each time interval, six replicates were used (*N* = 6)

^h^For each compound, the *p* value denotes a significant quantitative difference between non-sterile-filtered larval CHC trails of *T. confusum* 0 h, 24 h, and 48 h after trail deposition (one-way ANOVA or *Kruskal-Wallis* test, ns = not significant)

When comparing the chemical composition of sterile- and non-sterile-filtered trails for each time interval past trail deposition, we found that sterile-filtered trails were missing five compounds, which were detected in the non-sterile-filtered trails (ID XVII, XX, XXII, XXIII, and XXIV in Table 2). When statistically comparing the quantities of the compounds present in both CHC trail types, we found that at all time intervals the quantity of individual CHCs was significantly higher in non-sterile-filtered trails than in sterile ones (for *p*-values see Table S3). Only the quantities of *n*-C25 and *n*-C27 (ID I and VIII in Table S3) did not significantly differ between both CHC trail types at the respective time intervals.

### Change of Kairomonal Activity of Host Trails in the Course of Time

**Fig. 3:**
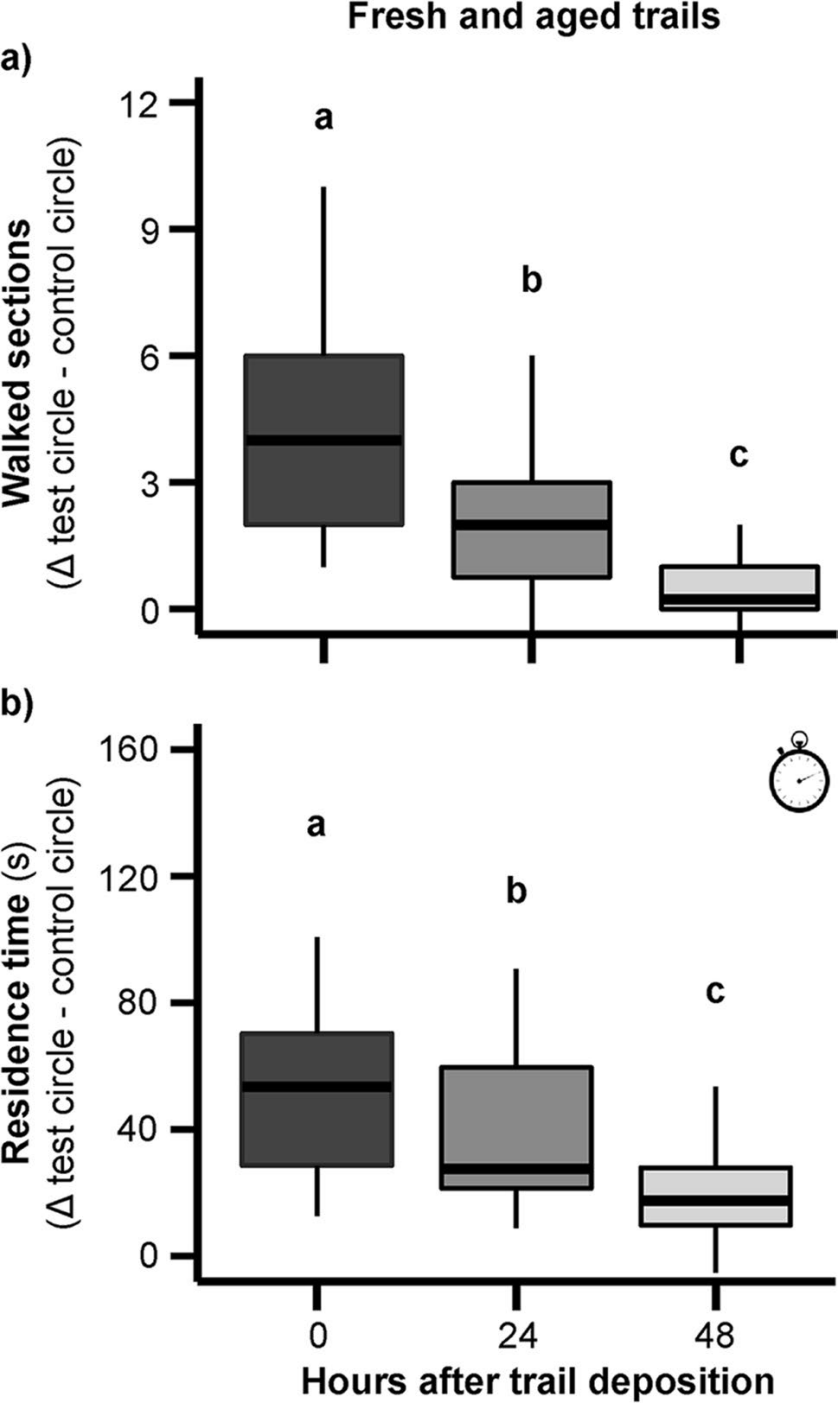
Behavioral response of female *Holepyris sylvanidis* to trails consisting of non-sterile-filtered larval extracts of *Tribolium confusum* (5 LE of *T. confusum* 4th instar larvae per trail). Circular trails were deposited 0 h, 24 h, or 48 h before being offered to the parasitoids (*N* = 28 per time interval). Mean differences (Δ) of **a)** walked sections and **b)** residence time (indicated by the clock inset) on test and control circle at different time intervals are displayed. Test circle: hexane-extracted larval host trails. Control circle: hexane only. The parasitoid’s trail-following activity at different time intervals was analyzed by a *Kruskal-Wallis* test followed by pairwise *Wilcoxon’s* rank-sum test with *Bonferroni-Holm* correction. Different letters indicate significant differences at *P* < 0.05

Our bioassays confirmed previous results of Fürstenau and Hilker (2017) and show a clear kairomonal activity of freshly laid CHC trails, but a decrease of the kairomonal effect within two days after trail deposition. Parasitoid females covered a significantly greater distance on freshly laid CHC trails than on “aged” 24 h- or 48 h-old ones (Fig. 3a). When comparing the walking distance on CHC-consisting test trails and (hexane) control trails, significantly greater distances were covered on CHC trails 0 h and 24 h after trail deposition, while this preference was lost 48 h after trail deposition (Table S4). When comparing the residence time on test and control trails, the residence time on test trails was always significantly higher than on control trails (Table S4). However, the time spent by the parasitoids on test trails decreased considerably with the “age” of a trail. Parasitoids spent significantly more time on freshly laid CHC trails than on 24 h- or 48 h-old ones (Fig. 3b).

**Fig. 4:**
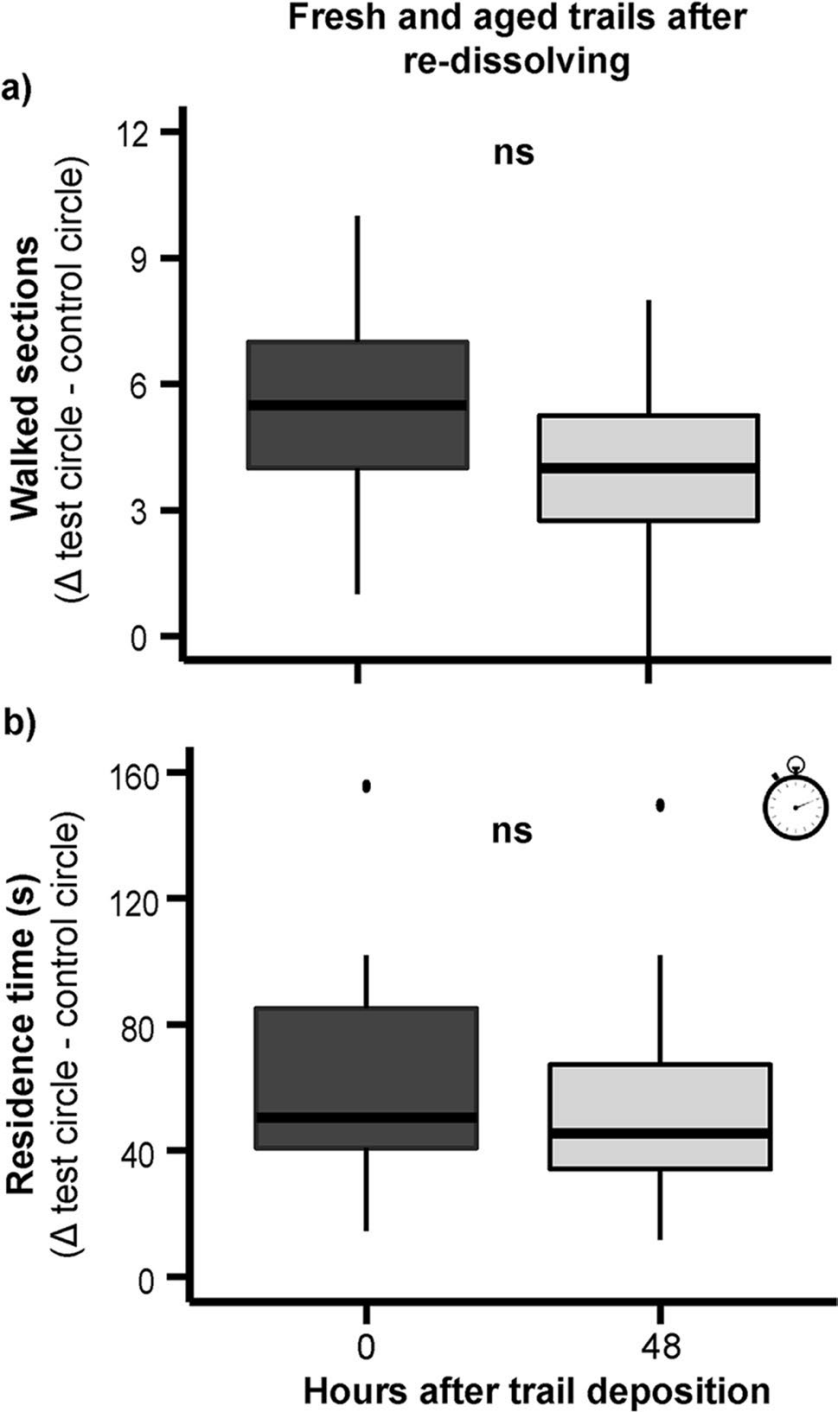
Behavioral response of female *Holepyris sylvanidis* to hexane-redissolved trails from non-sterile-filtered larval extracts of *Tribolium confusum* (5 LE of *T. confusum* 4th instar larvae per trail). The circular trails were offered to the parasitoids as freshly laid trails (0 h) or 48 h after deposition. Both trail types had been re-dissolved with n-hexane (25 μl) prior to the beginning of the bioassays (*N* = 28 per time interval). Test circle: hexane-extracted larval host trails. Control circle: hexane only. Mean differences (Δ) of **a)** walked sections and **b)** residence time (indicated by the clock inset) on test and control circle at different time points are displayed. The parasitoid’s trail-following activity at different time intervals was analyzed by *Student’s t*- test or *Wilcoxon’s* rank-sum test (ns = not significant, *P* > 0.05)

We further studied whether the kairomonal effect of 48 h-old larval trails could be reactivated by applying hexane to them. Parasitoids covered a similar walking distance and spent similar time on 48 h-old CHC trails with hexane reapplication as on freshly laid (0 h), untreated CHC trails (Fig. 4a, b). When comparing the parasitoid’s response to 48 h-old CHC trails with hexane reapplication to the hexane control trails, the results show that they covered a greater distance on re-dissolved 48 h-old test trails than on the controls and spent significantly more time on these trails (Table S4). Hence, an application of hexane fully restored the kairomonal activity of 48 h-old CHC trails.

### Change of Trail Structures in the Course of Time

We investigated by cryo-SEM whether trails obtained from hexane extracts of *T. confusum* larvae show changes in physical structures over time past trail deposition.

**Fig. 5:**
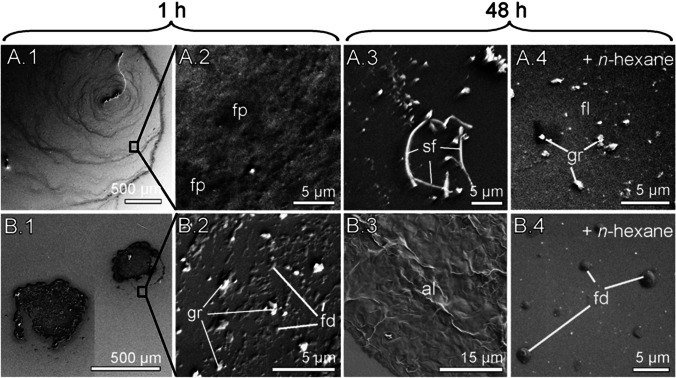
Cryo-SEM images of non-sterile-filtered hexane extracts of *Tribolium confusum* larvae applied to polar (A.1–4) and apolar (B.1–4) silicon wafers. Photos were taken at different time intervals after release onto the substrate, i.e. after 1 h (A.1–2, B.1–2) and 48 h (A.3–4, B.3–4). Note the “coffee ring” pattern in A.1, thin fluid patches in A.2, solidified filaments in A.3, very thin, re-dissolved fluid patches and granules in A.4, voluminous residues in B.1, tiny fluid droplets at the edges of larger patches in B.2, solidified amorphous films in B.3, and re-dissolved small fluid droplets in B.4. al, solidified, amorphous layer; fd, fluid droplets; fl, fluid layer; fp, fluid patches; sf, solidified filaments; gr, granules

Both on a polar and non-polar substrate, untreated trails changed their microscopically visible structures within 48 h (Fig. 5A1-A3, B1–3). One hour after application of the CHC trail onto the substrate, fluid patches or droplets were visible (Fig. 5A2, B2). On the polar substrate, the larval trail extract formed a ring-shaped pattern, which occurred 1 h after deposition and evaporation of hexane; such a formation has also been described as “coffee-ring effect” (Deegan et al. 1997, 2000). After 48 h, solidified filamentous structures were visible (Fig. 5A3). The filamentous structures were visible as clearly outlined, elongated threads on the polar substrate, while they were embedded in a solidified, amorphous layer with a gibbous surface on the apolar substrate (Fig. 5B3).

When applying hexane to a 48 h-old CHC trail, again fluid-like structures of these re-dissolved trails were visible, i.e. a fluid layer with granules on the polar substrate (Fig. 5A4) and droplets on the apolar substrate (Fig. 5B4).

## Discussion

Our present study addressed the question of whether the quick loss of kairomonal activity (within two days) of host larval trails used by a foraging larval parasitoid is due to chemical changes which might be caused by microbial activity. Our GC-MS analyses revealed that the chemical pattern of trails from *T. confusum* larval extracts (hereafter referred to larval trails) does not change over a period of 48 h after trail deposition, regardless of whether the possible microbial activity was excluded or not. Thus, no hints were detected (i) on chemical degradation of host CHC trails, (ii) on microbial contribution to a change in the chemical composition of the trails, and (iii) on the quantitative loss of CHCs due to e.g., evaporation. Our bioassays showed that the kairomonal activity of non-sterile CHC trails was restored by adding hexane as solvent to an inactive 48 h-old trail. These reactivated larval trails induced a comparable trail-following behavior in *H. sylvanidis* as we could observe with freshly laid trails. Cryo-SEM analysis showed that the CHC trails formed filamentous structures 48 h after the trail deposition, which were re-dissolved by hexane and then forming fluidic layers or droplets. Our results suggest that the quick loss of kairomonal activity of host larval trails after a short time is neither due to microbial degradation nor to a chemical change in the CHC profile, but due to a shift in the physical state and molecular packing of the CHC blend. The observed gradual assembling of host larval CHCs in filamentous structures after trail deposition might render the CHCs less accessible to the olfactory receptors by which insects perceive CHCs (Ozaki and Wada-Katsumata 2010). Such a reduced perceptibility is expected to reduce the kairomonal activity of the trails.

The physical states of individual, long-chained CHCs range from liquid to solid at ambient temperature. The CHC profile of *T. confusum* larvae is dominated by *linear* alkanes with a chain length from C25 to C31. These CHCs are known to form solid structures at ambient temperature and start melting at temperatures above 50 °C (Gibbs and Pomonis 1995; Maroncelli et al. 1982). Some long-chain *monomethyl-branched alkanes* can also solidify at room temperature range (Brooks et al. 2015), but the molecular packing and thus the melting behavior of this CHC type depends particularly on the position of the methyl group (Gibbs 1998). For example, 3-MeC25 becomes liquid at 40 °C, whereas 11-MeC25 does at approx. 20 °C. *Dimethyl-branched alkanes* show the lowest melting temperatures among all CHC types present on the cuticle of *T. confusum* larvae and are most likely liquid at room temperature (Gibbs 1998; Gibbs and Pomonis 1995).

The *T. confusum* larval extracts deposited as trails consisted of a blend of different CHC types with different physical states at ambient temperature. How might the physical state of this blend change within 48 h and lose its kairomonal activity?

We suggest the following scenario: After evaporation of the solvent hexane, the CHCs extracted from *T. confusum* host larvae began to rearrange themselves due to intermolecular interactions (e.g., van der Waals forces). These self-assembling processes might result in a more ordered state, and thus a change from liquid to solid occurred. The gradual decrease of the kairomonal activity of the larval trails suggests that the CHCs first formed a solid-liquid matrix in which some components had already solidified, whereas others were still present as liquids and therefore perceivable for *H. sylvanidis.* In the course of time, the CHC solidification might have gradually proceeded, thus rendering those CHCs informative to the parasitoid in their liquid phase no longer efficiently perceivable. Consequently, the CHCs lost the ability to induce trail-following behavior in the parasitoid.

Liquid CHCs are expected to have a greater interorganismic, informative relevance than solid ones because of their higher vapor pressure (Menzel et al. 2019; Othmer and Conwell 1945). Thus, they might not only be perceivable upon contact and bind to the odorant-binding proteins in the olfactory sensilla but could also be detected over some distance via the gas phase. For example, ants can perceive long-chain CHCs for differentiation of nestmates and non-nestmates without physical contact from a distance of 1 cm (Brandstaetter et al. 2008). This indicates that long-chain CHCs are volatile at least to a certain extent. Since *H. sylvanidis* females follow CHC trails of *T. confusum* host larvae by zig-zag movements along the trail, they most probably can directly contact the trail components; however, perception over a short distance cannot be excluded.

Due to their melting behavior, some mono- and dimethyl-branched CHCs are expected to be liquid in freshly laid larval trails at ambient temperatures, whereas other monomethyl-branched alkanes might begin to solidify right after trail deposition (Brooks et al. 2015; Gibbs and Pomonis 1995). The liquid aggregate state of methyl-branched alkanes might be relevant for eliciting trail-following behavior in *H. sylvanidis.* This suggestion is supported by our previous study, which showed that methyl-branched CHCs on the cuticle of *T. confusum* larvae are exploited by *H. sylvanidis* females for host recognition when directly contacting the host larvae (Awater-Salendo et al. 2020).

The sterile-filtered extracts were lacking some of those methyl-branched alkanes that were present in low quantities in the non-sterile trails, i.e. 3-MeC28, 5-MeC29, 5,X-DiMeC29, and 3,X-DiMeC29. We might have lost the low quantities of these compounds and *n*-C30 when filtering the larval extract through a sterile membrane. Despite the presence of these five compounds in non-sterile trails 24 h and 48 h after trail deposition, the kairomonal activity of these trails decreased significantly over time compared to freshly laid trails (= 0 h), and almost no trail-following behavior by parasitoid females was observed after 48 h. This suggests that these five long-chained CHCs are either not relevant in eliciting trail-following behavior of *H. sylvanidis* or they are no longer in a liquid phase at these time intervals past trail deposition, and thus not perceivable for the parasitoid.

Does also the substrate onto which *T. confusum* host larvae deposit their trails, affect the persistence of trail kairomonal activity? In the present study, we investigated host trails by applying larval cuticular extracts on inert glass ground, a polar substrate. This approach enabled us to determine possible shifts in the CHC profile over time since trails could be extracted from the glass for chemical analysis after distinct time intervals past deposition. Glass as substrate is very different from the natural substrates onto which *T. confusum* larvae release their trails. Larvae of this species are living where grains and further processed or refined plant and food products stored by humans are available. Thus, they release trails both onto polar substrates such as fine flour with all its carbohydrates, but also on whole grains with their often waxy, apolar surface. Our previous studies showed that the kairomonal activity of *T. confusum* trails naturally laid by larvae on course wheat grist (including seed coats, broken grain kernels) persisted as long as those trails extracted with hexane, i.e. 48 h past trail deposition (Fürstenau and Hilker 2017). Larval trails released by other insect species (coccinellid larvae, caterpillars) on natural substrates (leaves) are also known to elicit behavioral responses by braconid parasitoid species only for a maximum of two days past deposition. As in our study, these trails exclusively consist of long-chain CHCs with different structural features (Nakashima et al. 2004; Rostás and Wölfling 2009).

Under natural conditions, abiotic environmental factors might significantly influence the speed of transition from the liquid to solid state of CHCs. Especially temperature can significantly affect this transition (Gibbs 2002; Gibbs and Pomonis 1995). For instance, the different CHC profiles of several ant species became entirely liquid at temperatures ranging between 30 °C and 45 °C (Menzel et al. 2019). Hence, it is likely that high temperatures retard the solidification process of CHCs of *T. confusum* larvae. If so, the larval trails would remain longer in the liquid state, and thus their kairomonal activity for parasitoids might last longer. Low temperatures, in contrast, have an opposite effect and accelerate solidification of CHCs. Accelerated solidification *T. confusum* CHCs possibly driven by low temperatures is expected to result in an earlier loss of the kairomonal activity of trails within two days at room temperature. We suggest that differences in ambient temperature lead to different behavioral responses of parasitoids to host trails consisting of CHCs.

If the loss of the informative activity of insects CHCs is caused by the solidification of the CHC blend, as suggested above for *T. confusum* trails, the question arises what keeps CHCs liquid or in a liquid-solid phase on the insect cuticle so that they are released in this physical state onto a substrate. Our study suggests that compounds not detectable in hexane extracts of insect CHCs are relevant for the physical state of CHCs on the insect’s cuticle. Proteins in an aqueous medium are candidate compounds. They are known to function as CHC carriers, transporting the apolar CHCs through an aqueous medium from their synthesis site (e.g., the oenocytes) to the outer layer of the cuticle (Mohammadzadeh-K et al. 1969; Schal et al. 1998; Wigglesworth 1990). In addition, it is known for insect footprints, i.e. adhesion-mediating tarsal secretion, that they are formed by nanodroplets containing an apolar and a polar phase (Betz 2003; Hasenfuss 1977; Vötsch et al. 2002). The apolar phase often consists of a chemical blend whose hydrocarbon composition is often similar to that of the epicuticular grease, whereas water-soluble carbohydrates and proteins are assumed to be components of the polar phase (Geiselhardt et al. 2009, 2010; Gerhardt et al. 2016; Vötsch et al. 2002). The presence of proteinaceous components has been confirmed for tarsal fluids by Vötsch et al. (2002) and Betz et al. (2016). Hence, proteins might be involved in processes, which prevent CHCs from adopting a folded conformation and from forming densely packed assemblages by intermolecular interactions. After deposition of a blend of CHCs and proteins onto a substrate, proteins would quickly denature, thus allowing the CHCs to interact. To further elucidate the cause of the loss of the kairomonal activity of larval trails, future studies should focus on both the polar and apolar chemical composition of the epicuticular grease of host larvae and their naturally laid trails.

Taken together, our study showed that the CHC blend of an insect trail does neither qualitatively nor quantitatively change within 48 h past trail deposition but loses its kairomonal activity for a parasitoid within this time interval. This result was independent of whether microbial degradation of trails had been excluded or not. Since a change in the CHC composition of the trail cannot be made responsible for the temporary kairomonal effect, our study rather suggests that CHCs present in their liquid phase in freshly laid host trails gradually form solid structures and thus become less perceptible (as host-indicating cues) for foraging parasitoids. Future studies need to further elucidate whether as yet unconsidered polar compounds contribute to the gradual loss of kairomonal activity of host larval CHC trails.

## Supplementary Information

ESM 1 (PDF 139 kb)

## Data Availability

Datasets generated in the current study are available upon request via the corresponding author.

## Code Availability

Coding in “R” used in the current study is available upon request via the corresponding author.
