# Supplementary material for: Cuticular Hydrocarbon Trails Released by Host Larvae Lose their Kairomonal Activity for Parasitoids by Solidification
Source: J Chem Ecol. 2021 Sep 16;47(12):998–1013. doi: 10.1007/s10886-021-01310-w (PMC8642257; doi:10.1007/s10886-021-01310-w)
Supplement: Supplementary file 1 — (PDF 139 kb) [file 10886_2021_1310_MOESM1_ESM.pdf]

ELECTRONIC SUPPLEMENTARY MATERIAL

CUTICULAR HYDROCARBON TRAILS RELEASED BY HOST LARVAE LOSE THEIR KAIROMONAL ACTIVITY FOR PARASITOIDS BY SOLIDIFICATION

SARAH AWATER-SALENDO[1,2], DAGMAR VOIGT[3], MONIKA HILKER[2], BENJAMIN FÜRSTENAU[1*]

[1] *Julius Kühn Institute, Federal Research Centre for Cultivated Plants, Institute for Ecological Chemistry, Plant Analysis and Stored Product Protection, Königin-Luise-Str.19, 14195 Berlin, Germany*

[2] *Dahlem Centre of Plant Science, Institute of Biology, Applied Zoology/Animal Ecology, Freie Universität Berlin, Haderslebener Str.9, 12163 Berlin, Germany*

[3] *Institute of Botany, Faculty of Biology, Technische Universität Dresden, Zellescher Weg 20b, 01217 Dresden, Germany*

Journal of Chemical Ecology

* Author for correspondence (e-mail: benjamin.fuerstenau@julius-kuehn.de)

Table S1 Results of the SIMPER analysis comparing the chemical composition of sterile-filtered hexane extracts of *Tribolium confusum* larvae 0 h, 24 h or 48 h after trail (extract) deposition (overall average dissimilarity index = 5.35)

Compound	RI_{cal}[a]	Average dissimilarity	Contribution (%)	Cumulative (%)	Hours after trail deposition[b] 0	24	48
n-C27	2700	1.48	27.71	27.71	45.10	45.50	46.70
n-C25	2498	1.09	20.29	48.01	17.20	17.10	17.60
n-C29	2897	0.63	11.79	59.80	9.99	10.00	9.71
11-/13-MeC27	2730	0.42	7.80	67.60	6.95	6.91	6.91
5-MeC27	2747	0.26	4.79	72.38	3.28	3.25	3.11
12-/13-/14-MeC28	2828	0.21	3.86	76.24	0.81	1.02	0.56
n-C28	2797	0.20	3.82	80.06	4.11	4.11	4.03
3.X-DiMeC27	2803	0.18	3.29	83.35	2.28	2.04	1.97
5.X-DiMeC27	2778	0.16	2.95	86.30	1.69	1.64	1.50
11-/13-MeC29	2928	0.14	2.61	88.92	1.07	1.06	0.78
n-C26	2598	0.12	2.22	91.14	2.58	2.45	2.48
4-MeC28	2854	0.10	1.89	93.03	0.47	0.46	0.30
3-MeC27	2771	0.09	1.75	94.78	2.97	2.94	2.89
3-MeC29	2970	0.07	1.27	96.05	0.20	0.23	0.27
10-/11-/12-/13-MeC26	2633	0.06	1.05	97.09	0.46	0.44	0.41
3-MeC25	2573	0.05	1.00	98.09	0.33	0.33	0.32
4-MeC26	2656	0.05	0.85	98.94	0.23	0.21	0.18

n-C31	3096	0.02	0.44	99.39	0.12	0.07	0.08
11-/13-MeC25	2533	0.02	0.41	99.80	0.11	0.12	0.12
5-MeC25	2550	0.01	0.20	100.00	0.07	0.07	0.08
			99.99	100.00	100.02	99.95	100.00

[a] Retention index calculated on a HP-5ms capillary column (30 m x 0.25 mm x 0.5 µm)

[b] Relative quantities are given as % LE^{-1}, N = 5 per time interval

Table S2 Results of the SIMPER analysis comparing the chemical composition of non-sterile-filtered hexane extracts of *Tribolium confusum* larvae 0 h, 24 h or 48 h after trail (extract) deposition (overall average dissimilarity index = 4.68)

Compound	RI_{cal}[a]	Average dissimilarity	Contribution (%)	Cumulative (%)	Hours after trail deposition[b]		
					0	24	48
n-C27	2708	1.04	23.55	23.55	31.50	31.30	30.70
11-/13-MeC27	2735	0.58	13.06	36.61	8.68	8.86	8.62
n-C25	2503	0.48	10.88	47.48	11.30	11.40	11.30
5-MeC27	2750	0.41	9.37	56.85	4.20	4.24	4.85
n-C29	2904	0.29	6.51	63.36	16.20	16.50	16.30
11-/13-MeC29	2930	0.22	4.95	68.31	2.49	2.38	2.40
n-C28	2801	0.19	4.30	72.61	5.84	6.03	5.99
5,X-DiMeC27	2781	0.13	2.84	75.45	2.09	2.03	1.97
3-MeC27	2774	0.13	2.83	78.29	4.07	4.21	4.13
n-C26	2599	0.11	2.45	80.74	2.90	2.95	3.01
3,X-DiMeC27	2806	0.10	2.29	83.02	2.83	2.82	2.82
12-/13-/14-MeC28	2830	0.09	2.11	85.14	1.09	1.09	1.09
10-/11-/12-/13-MeC26	2633	0.09	1.95	87.08	0.80	0.80	0.88
3-MeC28	2873	0.07	1.51	88.60	0.39	0.26	0.33
4-MeC28	2856	0.06	1.31	89.91	0.83	0.76	0.79
3,X-DiMeC29	3004	0.06	1.30	91.21	0.38	0.32	0.36
n-C31	3098	0.06	1.29	92.50	0.73	0.68	0.70
4-MeC26	2658	0.05	1.24	93.74	0.44	0.39	0.48
11-/13-MeC25	2534	0.05	1.12	94.86	0.31	0.27	0.37
3-MeC25	2573	0.05	1.05	95.92	0.73	0.71	0.75
3-MeC29	2972	0.04	0.98	96.90	0.78	0.76	0.79
5-MeC29	2948	0.04	0.96	97.86	0.50	0.41	0.41
5,X-DiMeC29	2979	0.04	0.90	98.76	0.46	0.38	0.41
5-MeC25	2550	0.03	0.71	99.48	0.20	0.18	0.22
n-C30	2999	0.02	0.52	100.00	0.35	0.32	0.34
			100.01		100.09	100.05	100.01

[a] Retention index calculated on a HP-5ms capillary column (30 m x 0.25 mm x 0.5 μm)

[b] Relative quantities are given as % LE^{-1}, N = 6 per time interval

Table S3 Comparison of the chemical composition of sterile-filtered and non-sterile-filtered hexane extracts of *Tribolium confusum* larvae 0 h, 24 h or 48 h after trail (extract) deposition

			**Hours after trail deposition**[c]								
			**0**[d]			**24**[d]			**48**[d]		
			**Non-sterile**	**Sterile**		**Non-sterile**	**Sterile**		**Non-sterile**	**Sterile**	
**Compound**[a]	**ID**	**RI_{cal}**[b]	Mean ± SE (ng)	Mean ± SE (ng)	***P***[e]	Mean ± SE (ng)	Mean ± SE (ng)	***P***[e]	Mean ± SE (ng)	Mean ± SE (ng)	***P***[e]
n-C25	I	2498	20.12 ± 0.67	20.52 ± 3.04	ns	19.47 ± 0.77	17.99 ± 3.53	ns	19.10 ± 0.49	21.83 ± 2.40	ns
11-/13-MeC25	II	2533	0.56 ± 0.06	0.13 ± 0.03	***	0.46 ± 0.03	0.13 ± 0.03	***	0.63 ± 0.10	0.15 ± 0.03	**
5-MeC25	III	2550	0.36 ± 0.04	0.08 ± 0.02	***	0.31 ± 0.02	0.08 ± 0.02	***	0.38 ± 0.06	0.10 ± 0.01	**
3-MeC25	IV	2573	1.31 ± 0.12	0.40 ± 0.09	***	1.22 ± 0.06	0.36 ± 0.10	***	1.28 ± 0.07	0.40 ± 0.08	***
n-C26	V	2598	5.19 ± 0.23	3.10 ± 0.43	**	5.02 ± 0.15	2.61 ± 0.54	**	5.12 ± 0.15	3.07 ± 0.34	***
10-/11-/12-/13-MeC26	VI	2633	1.44 ± 0.16	0.53 ± 0.04	**	1.37 ± 0.09	0.46 ± 0.11	***	1.50 ± 0.15	0.51 ± 0.09	***
4-MeC26	VII	2656	0.79 ± 0.08	0.27 ± 0.03	***	0.66 ± 0.05	0.21 ± 0.06	***	0.82 ± 0.10	0.22 ± 0.05	***
n-C27	VIII	2700	56.50 ± 2.77	53.27 ± 5.70	ns	53.37 ± 1.68	46.66 ± 7.15	ns	52.23 ± 0.78	57.52 ± 5.41	ns
11-/13-MeC27	IX	2730	15.75 ± 1.56	8.10 ± 0.75	**	15.13 ± 0.90	7.25 ± 1.51	**	14.72 ± 0.95	8.61 ± 1.12	**
5-MeC27	X	2747	7.61 ± 0.65	3.81 ± 0.32	***	7.24 ± 0.34	3.35 ± 0.65	***	8.30 ± 1.19	3.84 ± 0.45	**
3-MeC27	XI	2771	7.30 ± 0.30	3.50 ± 0.39	***	7.17 ± 0.10	3.07 ± 0.59	**	7.02 ± 0.16	3.58 ± 0.38	***
5,X-DiMeC27	XII	2778	3.79 ± 0.35	1.95 ± 0.15	**	3.47 ± 0.19	1.71 ± 0.37	**	3.36 ± 0.18	1.86 ± 0.27	***
n-C28	XIII	2797	10.45 ± 0.41	4.86 ± 0.53	***	10.28 ± 0.18	4.23 ± 0.71	***	10.18 ± 0.21	4.97 ± 0.50	***
3,X-DiMeC28	XIV	2803	5.10 ± 0.35	2.64 ± 0.18	***	4.82 ± 0.16	2.15 ± 0.49	**	4.80 ± 0.23	2.42 ± 0.31	**
12-/13-/14-MeC28	XV	2828	1.98 ± 0.19	0.93 ± 0.07	**	1.86 ± 0.12	1.07 ± 0.32	*	1.87 ± 0.18	0.70 ± 0.14	***
4-MeC28	XVI	2854	1.50 ± 0.12	0.54 ± 0.03	***	1.29 ± 0.05	0.47 ± 0.13	***	1.35 ± 0.11	0.36 ± 0.07	***
3-MeC28	XVII	2873	0.71 ± 0.16			0.44 ± 0.02			0.57 ± 0.08		
n-C29	XVIII	2897	29.10 ± 1.57	11.77 ± 1.28	***	28.06 ± 0.52	10.42 ± 1.86	***	27.68 ± 0.33	11.96 ± 1.20	***
11-/13-MeC29	XIX	2928	4.51 ± 0.43	1.25 ± 0.12	***	4.06 ± 0.32	1.15 ± 0.29	***	4.10 ± 0.39	0.93 ± 0.09	**
5-MeC29	XX	2948	0.91 ± 0.11			0.70 ± 0.02			0.71 ± 0.05		
3-MeC29	XXI	2970	1.40 ± 0.10	0.23 ± 0.05	***	1.29 ± 0.05	0.27 ± 0.11	**	1.34 ± 0.09	0.31 ± 0.06	***
5,X-DiMeC29	XXII	2979	0.83 ± 0.05			0.65 ± 0.03			0.70 ± 0.06		
n-C30	XXIII	2999	0.64 ± 0.03			0.54 ± 0.03			0.58 ± 0.05		
3,X-DiMeC29	XXIV	3004	0.68 ± 0.07			0.55 ± 0.04			0.61 ± 0.12		
n-C31	XXV	3096	1.29 ± 0.04	0.13 ± 0.01	**	1.15 ± 0.10	0.08 ± 0.04	***	1.19 ± 0.05	0.10 ± 0.01	***

[a] *n*-alkanes were identified by comparing RIs and mass spectra with authentic standards. Methyl-branched alkanes were tentatively identified by the diagnostic ions, which resulted from favored fragmentation at branched points (see Fürstenau and Hilker 2017) and by comparing RIs with data from literature

[b] Retention index calculated on a HP-5ms capillary column (30 m x 0.25 mm x 0.5 µm)

[c] For the preparation of host larval trails, see experimental part

[d] N = 5-6 per trail type and time interval

[e] For each compound, the *p*-value denotes significantly quantitative differences between non-sterile and sterile-filtered larval CHC trails of *T. confusum* 0 h, 24 h or 48 h after trail deposition (*Student's t*-test, *Welch's t*-test or *Wilcoxon's* rank-sum test, ns = not significant, * $P < 0.05$, ** $P < 0.01$, *** $P < 0.001$)

Table S4 Behavioral response of female *Holepyris sylvanidis* to trails consisting of non-sterile-filtered hexane extracts of *Tribolium confusum* larvae; the response to trails was tested **a)** 0 h, 24 h, and 48 h after trail deposition (no further treatment of trails), or **b)** after application of *n*-hexane to freshly laid trails (0 or 48 h-old trails (re-dissolved larval trails) just prior to testing. CHC test trail: host larval extracts. Control trail: hexane only. The trail-following activity was evaluated based on (i) the residence time and (ii) the walked sections on a circular trail

		Residence time[b]		Walked sections[b]	
**Trails: Non-sterile filtered larval extracts of *T. confusum*** (5 LE per trail)[a]	**Time interval** (h)	**CHC trail** [c] Mean ± SE (s)	**Control trail** [c] Mean ± SE (s)	**CHC trail** [c] Mean ± SE (sections)	**Control trail** [c] Mean ± SE (sections)
**a)** Larval trails	0	67.36 ± 4.99[a]	14.72 ± 1.18[b]	5.39 ± 0.46[a]	1.14 ± 0.18[b]
	24	47.75 ± 4.89[a]	8.63 ± 1.08[b]	2.71 ± 0.33[a]	0.86 ± 0.18[b]
	48	28.62 ± 2.56[a]	9.14 ± 1.01[b]	1.25 ± 0.20[a]	0.82 ± 0.14[a]
**b)** Re-dissolved larval trails	0	73.16 ± 5.84[a]	11.46 ± 1.15[b]	6.21 ± 0.43[a]	1.04 ± 0.22[b]
	48	65.43 ± 5.43[a]	12.68 ± 1.17[b]	4.61 ± 0.36[a]	1.14 ± 0.20[b]

[a] For preparation of host larval trails, see experimental part

[b] Different letters indicate that the parasitoid's response to the control and CHC (test) trail differed significantly ($P < 0.001$, *Student's t*-test or *Wilcoxon's* signed-rank test for paired data)

[c] $N = 28$ per treatment and time interval
